# Impact of a parent-child sexual communication campaign: results from a controlled efficacy trial of parents

**DOI:** 10.1186/1742-4755-7-17

**Published:** 2010-07-21

**Authors:** Kevin C Davis, Jonathan L Blitstein, W Douglas Evans, Kian Kamyab

**Affiliations:** 1 Public Health Policy Research Program, RTI International, 3040 Cornwallis Road, Research Triangle Park, NC 27709, USA; 2 School of Public Health and Health Services, The George Washington University, 2175 K, Street NW, Washington, DC 20037, USA

## Abstract

**Background:**

Prior research supports the notion that parents have the ability to influence their children's decisions regarding sexual behavior. Yet parent-based approaches to curbing teen pregnancy and STDs have been relatively unexplored. The Parents Speak Up National Campaign (PSUNC) is a multimedia campaign that attempts to fill this void by targeting parents of teens to encourage parent-child communication about waiting to have sex. The campaign follows a theoretical framework that identifies cognitions that are targeted in campaign messages and theorized to influence parent-child communication. While a previous experimental study showed PSUNC messages to be effective in increasing parent-child communication, it did not address how these effects manifest through the PSUNC theoretical framework. The current study examines the PSUNC theoretical framework by 1) estimating the impact of PSUNC on specific cognitions identified in the theoretical framework and 2) examining whether those cognitions are indeed associated with parent-child communication

**Methods:**

Our study consists of a randomized efficacy trial of PSUNC messages under controlled conditions. A sample of 1,969 parents was randomly assigned to treatment (PSUNC exposure) and control (no exposure) conditions. Parents were surveyed at baseline, 4 weeks, 6 months, 12 months, and 18 months post-baseline. Linear regression procedures were used in our analyses. Outcome variables included self-efficacy to communicate with child, long-term outcome expectations that communication would be successful, and norms on appropriate age for sexual initiation. We first estimated multivariable models to test whether these cognitive variables predict parent-child communication longitudinally. Longitudinal change in each cognitive variable was then estimated as a function of treatment condition, controlling for baseline individual characteristics.

**Results:**

Norms related to appropriate age for sexual initiation and outcome expectations that communication would be successful were predictive of parent-child communication among both mothers and fathers. Treatment condition mothers exhibited larger changes than control mothers in both of these cognitive variables. Fathers exhibited no exposure effects.

**Conclusions:**

Results suggest that within a controlled setting, the "wait until older norm" and long-term outcome expectations were appropriate cognitions to target and the PSUNC media materials were successful in impacting them, particularly among mothers. This study highlights the importance of theoretical frameworks for parent-focused campaigns that identify appropriate behavioral precursors that are both predictive of a campaign's distal behavioral outcome and sensitive to campaign messages.

## Background

Early debut of sexual activity is associated with greater risk of HIV and other sexually transmitted diseases (STDs) as well as unwanted pregnancy among teens in the United States. While the prevalence of sexual intercourse and pregnancy among teens has declined significantly in the United States since the early 1990s [1,2], more recent data suggest that rates of teen pregnancy may be on the rise again [2]. Furthermore, the social and medical costs associated with STDs and teen pregnancy in the United States are among the highest in all developed countries [3-5]. To date, interventions for curbing teen pregnancy and the spread of STDs among teens have focused on educational programs for youth, many of which include sexual abstinence curricula.

While there is debate about the effectiveness of youth-focused abstinence education programs, prior research overwhelmingly supports the notion that parents have the ability to influence their children's decisions regarding sexual behavior, use of contraceptives, and disease prevention. Yet parent-based approaches to curbing teen pregnancy and STDs have been relatively unexplored. A number of studies show that parent-child communication about sex is related to delayed onset of sexual intercourse [6-12]; increased contraceptive use in daughters [12-14]; and increased disease prevention behaviors, including condom use and fewer sexual partners [6,11,15].

Given the lack of parent-based approaches to delaying sexual intercourse among adolescents, the U.S. Department of Health and Human Services launched the Parents Speak Up National Campaign (PSUNC). PSUNC is a multimedia social marketing campaign targeted to parents and is aimed at promoting parent-child communication about sex. The campaign is predicated on the body of research noted above that describes the influence of parental communication on adolescents' decisions regarding sexual behavior. Launched nationally in June 2007, the campaign targets parents of 10- to 14-year-old children. The campaign primarily uses televised public service announcements (PSAs) supplemented by radio, print, and outdoor advertising. PSUNC television advertisements feature age-appropriate youth letting their parents know that they want to talk to them about sex and that they should talk "early and often." The campaign PSAs are also designed for multiple racial/ethnic audiences, including ads targeted to a general audience and ads for African American, Hispanic, and Native American parents. The advertisements also promote the campaign's Web site, http://www.4parents.gov, which provides age-appropriate information and guidance to parents about talking to their children about sex.

The campaign is grounded in social cognitive theory, which predicts a chain of cognitive events that lead to behavioral outcomes and choices [16,17]. The PSUNC conceptual model, published elsewhere [18], specifically posits that increased parent-child communication will result from the promotion of effective communication behaviors and expectations about the impact of those behaviors. As such, the campaign's messages directly promote parents' self-efficacy and outcome efficacy to communicate with their children as well as normative beliefs about the age at which children should wait to have sex. These cognitive elements are purposefully embedded in specific messages within the campaign ads and include (1) parents' self-efficacy that they can effectively talk with their children about sex, (2) parents' outcome-efficacy that talking with their children will translate into reduced or delayed sexual activity and long-term social and health benefits for their children, and (3) social norms regarding the age to which children should wait to have sex. The PSUNC conceptual model hypothesizes that these are important cognitive factors that the campaign will impact to drive change in downstream parent-child communication behaviors.

Understanding these cognitive precursors provides information that can be used to refine and improve messaging for PSUNC specifically and for social marketing campaigns more generally. While many studies have examined the role of social cognitive factors in behavior change, this paper joins the small but important set of studies that have examined the cognitive factors that precede parent-child communication specifically. In a study of primarily African American father-son dyads, for example, both self-efficacy and outcome expectation played a role in increasing communication related to sexual behavior [19]. Self-efficacy was also found to be a significant predictor of communication around sexual behavior in mother-daughter dyads [20] and to enhance parent-child communication related to sexual abuse [21].

A preliminary evaluation study of PSUNC was conducted using a randomized controlled trial (RCT) [22]. The RCT design was chosen based on the PSUNC implementation strategy and inherent limitations of that strategy. As noted above, the campaign was implemented with PSAs that aired nationwide. PSAs, however, are predominantly aired during slots of advertising time that are donated by the television and radio stations airing them. These advertising time slots most often occur during late hours or programs that are not heavily viewed, making them likely choices for advertising donations to public service campaigns. Because PSAs most often appear during donated advertising times with sparse audiences, overall exposure to PSAs is very low. Therefore, using telephone surveys or other field-based data collection methods to measure parents' awareness of PSUNC was not feasible. Such surveys likely would not capture measurable levels of parent exposure to the campaign, leaving little basis on which to make comparisons of parent-child communication outcomes between those exposed and those not exposed to the campaign. As such, an RCT was chosen to explicitly control parent exposure to PSUNC and ensure sufficient statistical power to assess the impact of campaign exposure on parent-child communication outcomes.

Preliminary results from the RCT found that PSUNC was efficacious in increasing parents' initiation of conversations about sex with their children and in promoting use of the campaign Web site [22]. However, this study does not address how these effects manifest themselves through the PSUNC theoretical model described earlier. For example, is there evidence that the campaign generated this impact through the targeting of specific cognitive factors identified in the theoretical model that were hypothesized to influence parent-child communication? Fuller tests of this theoretical model for PSUNC have yet to be conducted.

Understanding how PSUNC influences the cognitive precursors identified in the campaign's theoretical model can help inform future parent-child communication campaign development in several ways. First, it is useful to determine whether the specific cognitive factors identified in the campaign's theoretical model are indeed associated with parent-child communication. This association speaks to the validity of the campaign strategy as outlined in the theoretical model (i.e., does the campaign target factors that are both sensitive to exposure to PSUNC messages and in fact likely to influence parent-child communication?). Second, it is useful to consider which cognitive precursors to parent-child communication are most influenced by the campaign. This facilitates further refinement of campaign messages to target outcomes that are most sensitive to campaign exposure.

The present study builds on the prior RCT for PSUNC to more specifically examine the relationship between parents' exposure to PSUNC messages and cognitive precursors that are targeted by the campaign and theorized to influence parent-child communication. Specifically, we use the RCT data to (1) test whether the cognitive precursors targeted by the campaign are predictive of parent-child communication, (2) examine the impact of exposure to PSUNC messages on those cognitive precursors, and (3) explore possible effects of heavier exposure to PSUNC (i.e., receiving more PSUNC messages) on each of those cognitive outcomes. We then discuss implications of these findings both for future PSUNC implementation specifically and more generally for overall development of parent-based social marketing campaigns that use theoretical models.

## Methods

### Data and Experiment Design

Our data come from the PSUNC Parent Efficacy Study, a randomized controlled experiment conducted with parents of 10- to 14-year-olds from the Knowledge Networks (KN) online panel. Established in 1999, the KN panel is the only online research panel that is based on probability sampling. The KN panel is recruited using random-digit-dial telephone surveys and is weighted to match U.S. Census demographic benchmarks. Individuals who do not have a computer and access to the Internet are provided MSN television service and free monthly Internet access. This allows coverage of both online and offline households in the United States.

For this study, we first identified all adult KN panelists living with at least one child between the ages of 10 and 14 years (N = 3,217). Mothers and fathers were sampled separately, and parents who did not participate were not replaced by a parent of the opposite gender. The parent and one child in the eligible age range were paired, and all study surveys referenced that one child after the parent consented to participate. A total of 2,439 parents (75.8%) responded to the study invitation and were eligible to participate. Among these, 1,969 parents (1,125 mothers and 844 fathers) completed the baseline survey in the fall of 2007. Only one parent per household participated in the study. Study sample sizes were determined by power analyses conducted prior to the study. Additional details on study recruitment and eligibility are provided elsewhere [22].

Parents were randomly assigned to treatment and control conditions, where treatment consisted of exposure to PSUNC advertisements and print materials and control consisted of no exposure to PSUNC messages. Random assignment was carried out by a standard randomization algorithm in the Knowledge Networks online sampling system that gave all participants equal chances of being placed in any of the experiment conditions. Because this study uses a self-administered survey, participants were not aware of the other experiment conditions to which they were not assigned. Mothers and fathers were randomized into their respective experiment conditions separately. All participants were surveyed at five points in time: (1) baseline, prior to message exposure, (2) 4 weeks post-baseline, (3) 6 months post-baseline, (4) 12 months post-baseline, and (5) 18 months post-baseline. Mothers were further randomized into treatment and booster (additional messages) conditions at 4 weeks post-baseline to assess the effects of additional PSUNC messages. A larger baseline sample of mothers (N = 1,125) was collected to accommodate this additional randomization of treatment condition mothers. We included the booster condition for mothers only based on two factors. First, it is well-known that mothers more often engage in sexual communication with their children compared to fathers and the PSUNC ads reflect this in their mother-centric messages. Second, study resources were limited and did not permit booster conditions for both mothers and fathers. Parents who were assigned to treatment conditions received exposure to PSUNC messages via online multimedia immediately following the baseline survey and prior to each of the 4-week and 6-month follow-up surveys.

Each parent that was assigned to an exposure condition read, viewed, and listened to a collection of multimedia stimuli from PSUNC. All materials were viewed online during each survey session. This included one 60-second television PSA called "Talk to Me" that shows adolescent children asking their parents to talk to them about waiting to have sex. The multimedia package also included one 60-second radio PSA and two print PSAs. Preceding the 6-week follow-up survey, mothers who were assigned to the booster condition reviewed the same set of multimedia materials plus two additional print PSAs, one additional 60-second radio PSA, and one additional 60-second television PSA. The additional television PSA, called "Muffinhead," showed adolescent children telling their parents that they will still remain a close family if they talk to them about waiting to have sex. In addition to viewing the ads online within the survey, all treatment condition participants were mailed a DVD containing all media materials for their specific condition and were asked to view them between survey sessions.

The study questionnaire, consent procedures, and human subjects protection protocols were reviewed and approved by the sanctioned Institutional Review Board of RTI International. This study was also reviewed and approved by the Federal Office of Management and Budget prior to recruitment of the first subject (OMB control #0990-0311).

### Measures

Parents completed a 64-item survey at each study time point. The survey was self-administered online by each parent participant from the KN panel. The survey consisted of questions on sociodemographic characteristics; knowledge, attitudes, and beliefs about parent-child communication; parent-child communication social norms and expectations; and media habits. Our analysis focuses on the impact of exposure to PSUNC messages on four primary measures of theorized cognitions related to parent-child communication: (1) social norms on waiting until older to have sex, (2) parent efficacy to talk to their child about sex, (3) short-term expectations about their child's response to parent communication about sex, and (4) long-term expectations about the impact of parent-child communication on their child's future success in life. Each outcome was measured as a multi-item summative scale or two-item index (Table 1). We also examined how each of these factors is related to parent-child communication, the primary behavioral outcome variable studied in Evans et al. [22]. Each of these measures is described in more detail below.

**Table 1:** Efficacy Study Outcome Variables

Scale/Index Items	Response Categories
**Wait Until Older Social Norm Index**	
To what age do you think boys should wait before being sexually active?	1 (until 14), 2 (until 16), 3 (until 18+), 4 (until married)
To what age do you think girls should wait before being sexually active?	1 (until 14), 2 (until 16), 3 (until 18+), 4 (until married)
**Efficacy to Practice Parent-Child Communication**	
How sure are you that you can always explain to your child why s/he should wait to be sexually active?	1 (not sure at all or very unsure) to 3 (completely sure or very sure)
How sure are you that you can always explain to your child how to make a boy/girl wait until ready to be sexually active?	1 (not sure at all or very unsure) to 3 (completely sure or very sure)
How sure are you that you can always explain to your child how to tell a boy/girl no if your child does not want to be sexually active?	1 (not sure at all or very unsure) to 3 (completely sure or very sure)
How sure are you that you can always explain to your child ways to have fun with a boy/girl without being sexually active?	1 (not sure at all or very unsure) to 3 (completely sure or very sure)
**Short-term Outcome Expectation Scale**	
If you talk early and often with your child about sex, your child will...	
Be less likely to be sexually active as a young teen	1 (strongly disagree) to 4 (strongly agree)
Not think you are judgmental	1 (strongly disagree) to 4 (strongly agree)
Understand the benefits of waiting to have sex	1 (strongly disagree) to 4 (strongly agree)
Not listen to what you say	1 (strongly agree) to 4 (strongly disagree)
Think you are a hypocrite	1 (strongly agree) to 4 (strongly disagree)
Rebel and want to engage in sexual activity even more	1 (strongly agree) to 4 (strongly disagree)
**Long-term Outcome Expectation Index**	
By effectively talking with your child about delaying sexual activity, you will be able to positively impact your child's future success and happiness	1 (strongly disagree) to 4 (strongly agree)
If you can convince your child to wait to have sex, s/he will have a better chance to succeed as an adult	1 (strongly disagree) to 4 (strongly agree)

#### Social norms for delay of sexual initiation

This measure included two items that asked participants their views regarding how long a child should wait before becoming sexually active; one item asked about boys, and the other asked about girls. Response options were "until they are 12," "until they are 14," "until they are 16," "until they are 18," "until they are 21," and "until they are married." Principal factor analyses indicated that both items loaded strongly onto a single factor (Eigen value = 1.69) with high reliability (alpha = 0.94).

#### Efficacy to talk to child about sex

Four items were used to assess participants' belief in their ability to communicate with their child about sexual activity. Each item begins with the stem, "How sure are you that you can always explain to your child..." Seven category response options ranged from "completely sure" to "not at all sure" for each item. These items loaded strongly into a single factor (Eigen value = 1.81) with good reliability (alpha = 0.78).

#### Short-term outcome expectations

Six items ask participants to consider the immediate impacts of parent-child communications about sex. Each item begins with the stem, "If you talked early and often with your child about sex, your child will..." and concludes with statements about the child's behavior (e.g., be less likely to be sexually active as a teen) and the child's perception of the parent (e.g., think you are a hypocrite). Four category response options ranged from "strongly agree" to "strongly disagree" with no neutral option. Principal factor analyses showed these items loaded into a single factor (Eigen value = 1.60) with acceptable reliability (alpha = 0.70).

#### Long-term outcome expectations

Two items ask participants to consider whether delaying sexual initiation would have a positive impact on their children's future. Four category response options ranged from "strongly agree" to "strongly disagree" with no neutral option. These two items loaded into a single factor (Eigen value = 0.83) with adequate reliability (alpha = 0.69).

For each scale/index described in Table 1 we constructed relative change scores for each of the baseline to follow-up time periods (4 weeks, 6 months, 12 months, and 18 months). These were calculated by subtracting the baseline value of the outcome from the follow-up value and then dividing by the baseline value. The use of relative change scores helps account for the individual's baseline level for each outcome and potential correlation between the baseline and follow-up values [23].

#### Parent-child communication

To examine the relationship between each of the cognitive variables described above and parent-child communication behavior, we focus on actual parent recommendations to the child to wait to have sex, which was found to be significantly impacted by exposure to PSUNC messages in Evans et al. [22]. This measure is derived from the survey item that asks parents "Have you asked (recommended) that [child name] wait to have sex?" We created a dichotomous indicator for whether the parent answered "yes" to this question.

#### Control Variables

The study survey also included measures for a number of parent characteristics and other potential correlates of parent-child communication that we controlled for in our statistical analyses. These included parent marital status; educational attainment; race/ethnicity; age; full-time employment status; family structure (one or two parents in home); metropolitan statistical area (MSA) category (urban or rural); child gender; child's access to television, Internet, and other media in their bedroom; and an 8-item scale for parent involvement. The parent involvement scale includes items drawn from previous research [24,25] that measure joint parent-child activities and the frequency of those activities. The specific items in the parent involvement scale measure past month frequency ("never," "less often," "at least once a month," or "at least once a week") of (1) shopping, (2) going to the movies or sporting events, (3) watching television, (4) attending religious services, (5) doing homework, (6) attending a party, (7) volunteering, and (8) playing a game or a sport. Prior research shows that these items load into a single scale that has good reliability [22].

We also created a measure to account for potential contamination of the control condition. Although it was expected that natural exposure to PSUNC would be very low due to the limited reach of PSAs, there is still a small chance that participants in the control condition may be exposed to the campaign as it aired in the real world. The study instrument thus included a specific question on general awareness of PSUNC messages that asked "Have you ever seen or heard ads on television or radio with the Parents Speak Up National Campaign theme or slogan?" Participants could answer "yes" or "no" to this question. This variable was analyzed to assess the extent of possible control condition contamination and was included in our multivariable analyses to account for potential contamination.

### Statistical Analysis

#### Scale Reliability of Cognitive Precursors

We conducted principle factor analysis to assess reliability of each of the four cognitive outcomes examined in this study. The factor analysis was performed using the principal factor method in Stata statistical software. Scale and index reliability were then estimated using Cronbach's alpha coefficient for the scale, a measure of the internal consistency of the scale determined by the average inter-item correlation and the number of items in the scale [26,27]. We estimated Cronbach's alpha overall and separately for mothers and fathers in the study. Principal factor analyses indicated that the items within each of the four primary cognitive outcome scales/indices that we examined load into a single scale or index with factor loadings ranging from 0.38 to 0.92 for each item and Cronbach's alphas ranging from 0.69 to 0.94 for each scale/index.

#### Relationship between Cognitive Precursors and Parent-Child Communication

To test whether the cognitive factors targeted by the campaign are predictive of parent-child communication, we estimated a multivariable logistic regression that links the cognitive variables to future parent-child communication in a longitudinal framework. This analysis helps to assess the overall validity of the campaign strategy as outlined in the theoretical model (i.e., does the campaign target cognitive precursors that are in fact likely to influence parent-child communication?). Specifically, we estimated the odds of parent recommendation to wait to have sex at the 18-month follow-up as a function of each of the four cognitive variables at baseline. This model included a control variable for whether the parent had already made recommendations to wait at baseline. We also included control variables for whether the parent was in the study treatment or control conditions as well as baseline covariates for each of the individual control variables described earlier.

#### Impact of PSUNC on Cognitive Precursors

We used multivariable linear regressions to estimate the relationship between each time-specific change score and parent exposure to treatment conditions. We estimated separate models for each treatment effect at 4 weeks, 6 months, 12 months, and 18 months post-baseline. All control variables described earlier were included in each model. Because so little is known about father-child communication about sexual activity, we also estimated each model separately for mothers and fathers. All models were estimated using Stata Version 9 (College Station, Texas).

## Results

Study sample sizes, by experiment condition and survey wave, are summarized in Table 2. A total of 1,969 parents (1,125 mothers and 844 fathers) completed the baseline survey. Between the baseline and 18-month follow up survey, overall attrition was 49.9%. Analysis of attrition by experiment condition suggests that both mothers and fathers in the treatment conditions were slightly more likely to drop out of the study after 18 months. Among mothers, 47.3% of control condition respondents dropped out after 18 months, whereas 53.1% of treatment condition respondents dropped out. Among fathers, 18-month attrition rates were 42.1% within the control condition and 52.4% within the treatment condition. We further examined attrition by a number of demographic characteristics and found that 18-month attrition rates were not significantly different by parent race/ethnicity, education, or employment status. We further compared all study outcome variables as well as all baseline control variables that were included in our multivariable models by attrition. There were no statistically significant differences in any of the study outcome variables or baseline control variables between those who completed all 5 waves of the study and those who dropped out before completing all waves.

**Table 2:** Efficacy Experiment Sample Sizes

	Survey Sample Sizes				
Experiment Condition	Baseline	4-Week Follow-Up	6-Month Follow-Up	12-Month Follow-Up	18-Month Follow-Up
Mothers					
Control	349	326	270	233	184
Normal Treatment	776	663	266	219	175
Booster Treatment	--	--	275	220	189
					
Fathers					
Control	340	321	280	230	197
Normal Treatment	504	444	365	297	240
					
Total	1,969	1,754	1,456	1,199	985

*Note*: Normal treatment = Exposed to core PSUNC messages; Booster treatment = Exposed to core plus additional PSUNC messages.

Baseline distributions of each outcome variable are shown in Table 3. With the exception of the short-term expectation scale, the sample is generally clustered in the upper range of each outcome variable. However, significant proportions of both the mother and father samples are below outcome ceilings at baseline, indicating significant potential for change over time. Our use of relative change scores in our multivariable models allows for the inclusion of those already at outcome ceilings at baseline, capturing the treatment condition's protective effects in preventing relapse from the outcome ceilings.

**Table 3:** Baseline Outcome Variable Distributions

Outcome Variable	Mothers (N = 1,125)	Fathers (N = 844)
**Wait Until Older Social Norm Index**	%	%
2 (minimum)	0.00	0.26
3	0.00	0.00
4	1.22	3.17
5	1.94	2.25
6	47.09	50.33
7	3.36	5.55
8 (maximum)	46.38	38.44
**Efficacy to Practice Parent-Child Communication**		
6 (minimum)	0.10	0.40
7	0.20	0.13
8	6.52	9.96
9	7.64	10.36
10	12.83	15.14
11	15.89	15.54
12 (maximum)	56.82	48.47
**Short-Term Outcome Expectation Scale**		
9 (minimum)	0.10	0.00
10	0.00	0.00
11	0.10	0.00
12	0.10	0.54
13	0.31	0.54
14	0.92	0.54
15	1.74	2.95
16	3.90	4.26
17	8.51	10.98
18	21.44	25.57
19	15.28	15.80
20	12.51	12.85
21	10.15	7.90
22	8.00	6.29
23	6.46	5.09
24 (maximum)	10.46	6.69
**Long-Term Outcome Expectation Index**		
2 (minimum)	0.61	0.93
3	0.10	0.26
4	1.84	2.12
5	4.39	5.83
6	21.63	26.23
7	19.49	21.19
8 (maximum)	51.94	43.44

Parent sociodemographic characteristics are summarized in Table 4. Most parents were between the ages of 33 and 55, with a mean age of 43. The sample was predominantly white with low sample sizes of African American and Hispanic parents, relative to the U.S. population. The sample also contained a higher rate of college-educated parents compared with the United States as a whole. Child gender, child age, and parent employment status statistics resembled the U.S. population.

**Table 4:** Unweighted Sample Demographics of Efficacy Study Participants Who Completed All Five Survey Waves

TOTAL (*N *= 985)	Mothers (*N *= 548)			Fathers (*N *= 437)	
**Baseline Demographic Variable**	**Control (*N *= 184)**	**Normal Treatment (*N *= 175)**	**Booster Treatment (*N *= 189)**	**Control (*N *= 197)**	**Treatment (*N *= 240)**

Average parent age	43	42.8	41.6	44.9	44.7
Average child age	12.2	12.2	12.0	12.2	12.3
Parent education					
Less than high school	1.1%	1.7%	1.6%	3.1%	1.7%
High school graduate	14.7%	13.7%	10.1%	15.2%	13.3%
Some college	36.4%	40.6%	42.3%	30.0%	34.2%
Bachelors degree+	47.8%	44.0%	46.0%	51.8%	50.8%
Race/ethnicity					
White	86.4%	84.6%	85.7%	88.3%	90.0%
African American	7.1%	9.7%	7.9%	1.0%	3.8%
Hispanic	3.3%	4.0%	3.7%	3.1%	2.9%
Other	3.3%	1.7%	2.7%	7.6%	3.3%
Child gender					
Male	47.3%	49.1%	50.8%	60.9%	54.2%
Female	52.7%	50.9%	49.2%	39.1%	45.8%
Employment status					
Full-time	50.3%	44.8%	50.8%	85.6%	88.3%
Part-time	24.0%	24.4%	25.7%	3.1%	2.9%
Not employed	25.7%	30.8%	23.5%	11.3%	8.8%

*Note*: Five survey waves = baseline and 4 weeks, 6 months, 12 months, and 18 months post-baseline. Normal treatment = Exposed to core PSUNC messages; Booster treatment = Exposed to core plus additional PSUNC messages.

Multivariate logistic regressions of the relationship between campaign-targeted cognitive precursors at baseline and parent-child communication at 18 months post-baseline are shown in Table 5. Baseline norms favoring waiting until older to have sex were associated with parent recommendations to wait to have sex 18 months post-baseline among both mothers (OR = 1.21, p < 0.016) and fathers (OR = 1.15, p < 0.033). Expectations about long-term outcomes from parent-child communication were also associated with greater odds of parent recommendations to wait to have sex among both mothers (OR = 1.16, p < 0.017) and fathers (OR = 1.41, p < 0.001). Self efficacy to practice parent-child communication was not associated with follow-up parent recommendation to wait to have sex for either mothers or fathers. Short-term outcome expectations were only associated with parent-child communication at follow-up among fathers (OR = 1.13, p < 0.002).

**Table 5:** Multivariable Logistic Regression Showing Odds of Parent Recommendation to Wait to Have Sex at 18 Months Post-Baseline as a Function of PSUNC Cognitive Precursors at Baseline [95% Confidence Interval] (p-value)

	Outcome Variable: Parent Recommendation to Wait to Have Sex, 18 Months Post-Baseline	
**Baseline Independent Variables (Cognitive Precursors)**	**Mothers**	**Fathers**

**Wait Until Older Norm Index**	**1.21** **[1.04, 1.41] (0.016)**	**1.15** **[1.01, 1.31] (0.033)**
**Self-Efficacy Scale**	0.99 [0.88, 1.11] (0.804)	0.92 [0.82, 1.03] (0.134)
**Short-term Expectation Scale**	1.04 [0.97, 1.11] (0.323)	**1.13** **[1.04, 1.22] (0.002)**
**Long-term Expectation Index**	**1.16** **[1.03, 1.30] (0.017)**	**1.41** **[1.23, 1.61] (0.001)**

*Notes: *Model controls for the following variables measured at baseline: parent recommendation to wait to have sex (at baseline); child gender; parent marital status; highest educational attainment; race/ethnicity; parent age; employment status; family structure; whether child has computer, cable television, or Internet in his or her bedroom; treatment condition; metropolitan statistical area urban status; and parental involvement. Bold odds ratios are statistically significant at p < 0.05.

Results from the multivariate linear regression models of the relationship between exposure to PSUNC messages and these cognitive variables are summarized in Table 6. Among mothers, we found significant treatment effects at 4 weeks (b = 0.030, p < 0.001), 6 months (b = 0.037, p < 0.001), and 12 months (b = 0.028, p < 0.021) post-baseline for increased norms favoring waiting until older to have sex. For this outcome, we also found that mothers exposed to additional PSUNC messages in the booster condition exhibited greater change 6 months post-baseline in norms favoring waiting until older to have sex compared to mothers in the control condition (b = 0.027, p < 0.006). However, there was not a significant booster effect for this outcome relative to mothers in the normal treatment condition, suggesting that normal treatment (without additional PSUNC messages) was just as impactful as booster treatment exposure at 6 months post-baseline. No treatment effects for norms regarding the age to which parents' children should wait to have sex were observed at 18 months post-baseline.

**Table 6:** Multivariable Least Squares Regressions Showing Coefficients for Association between Changes in Parent-Child Communication Cognitions and Exposure to PSUNC [95% Confidence Interval] (p-value)

	Mothers				Fathers			
**Outcomes**	**4-Week Follow-up**	**6-Month Follow-up**	**12-Month Follow-up**	**18-Month Follow-up**	**4-Week Follow-up**	**6-Month Follow-up**	**12-Month Follow-up**	**18-Month Follow-up**

**Wait Until Older Norm Index**								
Normal Treatment	**0.030** **[0.02, 0.04] (0.001)**	**0.037** **[0.02, 0.06] (0.001)**	**0.028** **[0.01, 0.05] (0.021)**	0.009 [-0.02, 0.04] (0.487)	0.018 [-0.03, 0.04] (0.088)	0.007 [-0.02, 0.03] (0.592)	-0.006 [-0.04, 0.02] (0.685)	0.003 [-0.03, 0.04] (0.846)
Booster Treatment	--	**0.027** **[0.01, 0.05] (0.006)**	0.004 [-0.02, 0.03] (0.717)	0.021 [-0.01, 0.05] (0.122)	--	--	--	--
								
**Self Efficacy Scale**								
Normal Treatment	0.002 [-0.01, 0.02] (0.811)	**0.020** **[0.01, 0.04] (0.038)**	0.013 [-0.01, 0.04] (0.322)	-0.003 [-0.03, 0.03] (0.817)	0.015 [-.01, 0.04] (0.121)	0.010 [-0.01, 0.03] (0.443)	-0.002 [-0.03, 0.03] (0.895)	-0.006 [-0.04, 0.02] (0.687)
Booster Treatment	--	0.004 [-0.02, 0.02] (0.686)	-0.001 [-0.03, 0.03] (0.937)	0.005 [-0.02, 0.03] (0.736)	--	--	--	--
								
**Short-term Expectation Scale**								
Normal Treatment	0.006 [-0.01, 0.02] (0.394)	-0.017 [-0.04, 0.01] (0.084)	0.008 [-0.01, 0.03] (0.441)	-0.013 [-0.04, 0.01 (0.300)	0.006 [-0.01, 0.02] (0.456)	-0.006 [-0.02, 0.01] (0.478)	-0.024 [-0.05, 0.00] (0.060)	-**0.033** **[-0.06, -0.01] (0.008)**
Booster Treatment	--	**-0.021** **[-0.04, 0.00] (0.037)**	-0.009 [-0.03, 0.01] (0.396)	-0.016 [-0.04, 0.01] (0.196)	--	--	--	--
								
**Long-term Expectation Index**								
Normal Treatment	**0.034** **[0.01, 0.06] (0.004)**	**0.033** **[0.01, 0.06] (0.046)**	**0.036** **[0.01, 0.07] (0.043)**	0.004 [-0.04, 0.04] (0.848)	-0.002 [-0.03, 0.02] (0.861)	-0.012 [-0.04, 0.02] (0.440)	0.023 [-0.01, 0.06] (0.204)	-0.020 [-0.06, 0.02] (0.310)
Booster Treatment	--	**0.032** **[0.01, 0.06] (0.044)**	**0.042** **[0.01, 0.08] (0.024)**	0.006 [-0.04, 0.05] (0.815)	--	--	--	--

*Notes*: All models control for child gender; parent marital status; highest educational attainment; race/ethnicity; parent age; employment status; family structure; whether child has computer, cable television, or Internet in his or her bedroom; metropolitan statistical area urban status; parental involvement, and an indicator variable for prior exposure to PSUNC. Normal treatment = Exposed to core PSUNC messages; Booster treatment = Exposed to core plus additional PSUNC messages. Bold coefficients are statistically significant at p < 0.05.

We found evidence of a short-term normal treatment effect at 6 months post-baseline among mothers for increased parent efficacy to practice parent-child communication (b = 0.020, p < 0.038). No other treatment effects were observed for this outcome. Results further indicated a negative booster condition effect on short-term outcome expectations among mothers 6 months post-baseline. Booster treatment was associated with decreased expectations about short-term outcomes from parent-child communication at 6 months post-baseline (b = -0.021, p < 0.037) relative to mothers in the control condition. Exposure to PSUNC messages was not associated with either of these outcomes at 12 or 18 months post-baseline.

Among mothers, we found significant treatment effects at 4 weeks (b = 0.034, p < 0.004), 6 months (b = 0.033, p < 0.046), and 12 months (b = 0.036, p < 0.043) post-baseline for increased expectations about long-term outcomes from parent-child communication about sex. We also found significant booster treatment effects on this outcome among mothers. Booster treatment was associated with increased long-term outcome expectations at 6 months (b = 0.032, p < 0.044) and 12 months (b = 0.042, p < 0.024) post-baseline, relative to mothers in the control condition. However, the booster treatment effect was not significantly different compared to the normal treatment, suggesting normal treatment without additional messages was as effective as booster treatment with additional messages at each of the 6- and 12-month follow ups. Across all cognitive outcomes and follow-up time points, no positive treatment effects were observed for fathers.

## Discussion

PSUNC appears to be effective, under controlled conditions, in changing social norms regarding the age until which teens should wait to have sex and expectations about long-term outcomes from parent-child communication about sex. Specifically, mothers who were exposed to PSUNC in this experiment exhibited a larger change than control mothers in their norms toward the belief that teens should wait until they are older to have sex. Mothers who were exposed to PSUNC messages also exhibited larger increases than control mothers in their beliefs that parent-child communication about sex would have a positive impact on their child's future success. There is limited evidence of short-run effects among mothers on parent efficacy to communicate with their child. This effect surfaces at 6 months post-baseline and dissipates thereafter.

We also found some evidence of positive booster condition effects among mothers on social norms regarding age until which teens should wait to have sex and expectations about long-term parent-child communication outcomes. However, most of these effects were relative to mothers in the control condition. We generally did not find significant differences in effects between mothers that received the normal treatment condition and those that received the booster treatment. That is, in most instances where positive treatment effects occurred, the normal treatment without additional PSUNC messages appeared to be as effective as the booster treatment. Furthermore, all exposure effects observed in this study appear to dissipate after 12 months post-baseline. This pattern is consistent with experiment protocols for exposing treatment condition parents to PSUNC media prior to each of the 4-week and 6-month follow-up surveys. This may suggest that continual exposure is needed to sustain longer-term effects.

Results from our longitudinal model of parent-child communication confirm that the two cognitive factors most impacted by PSUNC messages (i.e., the "wait until older norm" and "long-term outcome expectations") are also strongly predictive of actual parent-child communication. We found that baseline measures of the "wait until older norm" and long-term outcome expectations are predictive of parent recommendations to wait at 18 months post-baseline. The other two cognitive variables targeted by the campaign and examined in this study (self-efficacy to communication and short-term expectations) were less associated with recommendations to wait. Combined, these results shed new light on how the effects of PSUNC on parent-child communication as reported in Evans et al. [22] manifest through the PSUNC theoretical model. That is, two of the specific cognitions that PSUNC appears to impact are also predictive of parent-child communication. The cognitions that PSUNC is not associated with are less predictive parent-child communication.

These findings have important implications for future PSUNC message development. First, our findings speak to the validity of the campaign's basic strategy that is outlined in its theoretical model. The campaign identified specific cognitive factors that were theorized to impact parent-child communication and then targeted those cognitive factors in its messages. Our results suggest that the "wait until older norm" and long-term outcome expectations were appropriate cognitions to target and the campaign was successful in impacting them. The "wait until older norm" is perhaps the most prominent cognition in all PSUNC messages, because it is implicit in almost every ad and campaign material. However, other cognitions identified in the theoretical model were not impacted by PSUNC messages and do not appear to be predictive of parent-child communication. This suggest that if future PSUNC messages are developed, the campaign may be well-served by focusing primarily on the "wait until older norm" and long-term outcome expectations in its messaging.

Our findings also have implications for the development of parent-child communication messages more generally. This study highlights the importance of developing a theoretical framework that identifies appropriate cognitive precursors that are both predictive of a campaign's distal behavioral outcome and sensitive to campaign messages. This type of framework provides a roadmap of specific cognitions and other precursors to behavior that should be emphasized directly in campaign messages. It is important that this framework be theory-driven to ensure that parent-child communication messages are designed to take action on factors most likely to influence campaign-targeted behavioral outcomes.

This study also provides insights into potential gender differences in message processing for parent-child communication campaigns. Specifically, no exposure effects on cognitive precursors were shown for fathers. This finding contrasts with Evans et al. [22], who found that among both mothers and fathers, exposure to PSUNC was associated with a higher odds of parent recommendations to their child to wait to have sex. The apparent disconnect between these findings may be partly explained by actual gender differences in message processing as well as gender foci in the PSUNC messages themselves. First, many of the PSUNC messages, particularly early advertisements such as "Muffinhead," are mother-centric. Mothers may also be more attentive to PSUNC messages. A number of studies find that mothers are significantly more involved in the sexual health education of their children compared with fathers [28-30]. Therefore, it is possible that mothers more actively process PSUNC messages that promote parent-child communication about sex. Fathers may also follow a different process between message exposure and behavioral parent-child communication. That is, fathers may be more likely to receive the message and turn directly to behavioral action and engage less in preceding social/cognitive contemplation. Such a process would suggest the hypothesis that PSUNC should have diminished effects on cognitive outcomes among fathers specifically. These are also important considerations for future message development, suggesting that gender-specific message tailoring may be appropriate for parent-child communication campaigns.

It should also be noted that while PSUNC was airing during our study period with a limited PSA distribution, we found very little evidence of control condition contamination. As expected, most participants in the control condition (96%) indicated no awareness of PSUNC television or radio ads, suggesting that contamination of the control condition with real-world exposure was very low. However, there was still a small percentage (4%) of control condition participants that did indicate some level of awareness of the PSUNC ads. This is consistent with levels of exposure that might be expected with a campaign based on PSA distributions. In our multivariable models of message effects, this variable was not significantly associated with any of the main outcome variables and none of the primary study findings were sensitive to the inclusion or exclusion of our variable for PSUNC awareness. This would suggest that our study findings are not biased by control condition contamination.

The study has a few limitations. First, we examined the effects of parent exposure to PSUNC in a controlled setting using an online survey, which may not reflect real-world conditions. Although the online experimental design provides high internal validity, the external validity of our study is limited because it does not assess the campaign within a field-based setting [31]. However, experimental efficacy studies are an emerging evaluation tool, particularly when media campaign timelines and other logistical considerations preclude the use of in-field evaluation designs [32]. Second, although the KN panel includes both Internet and non-Internet households and uses random-digit-dialing methodologies for recruitment, it may not perfectly represent the U.S. population of parents. Our sample was predominantly white with greater educational attainment compared with the U.S. population as a whole. This limits the ability to generalize the findings to the broader population of parents in the United States. Third, survey attrition is slightly higher among treatment condition parents than among control parents. This is somewhat expected given the greater time burden incurred by treatment condition parents who were exposed to experiment media stimuli and answered additional questions about the stimuli they viewed. However, we found that 18-month attrition rates were not significantly different by any of the baseline analytic control variables included in this study.

Finally, because our study utilized a self-administered online survey that most participants complete on their home computers, there is no way to confirm with absolute certainty that all treatment condition participants attentively viewed all of the PSUNC media materials. However, in addition to viewing the materials online, all treatment participants were mailed a DVD containing all media materials for the study. The study questionnaire included an additional item that asked participants whether they actually viewed all of the media materials on the DVD they received. Data based on this item show that 94% of all treatment condition participants reported viewing the DVD in its entirety. Combined with online viewing of the materials during the survey sessions, these data suggest that exposure fidelity was likely very good.

## Conclusions

In summary, this study offers empirical evidence on how PSUNC's impact on parent-child communication may manifest through its influence on cognitive precursors that are predictive of parent-child communication. However, additional questions remain. For example, more work is needed to determine what other factors, such as parent-child relationships, individual sociodemographic characteristics, and other variables, mediate the effects of PSUNC and whether these mediating processes vary by parent gender. Formal mediation analyses are needed to better address these questions. Furthermore, little is known about how parent-based communication programs translate into changes in outcomes among children of parents who receive messages like those disseminated by PSUNC. Confirmatory data from child self-reports of parent-child communication outcomes are needed to address this. Future evaluation studies for PSUNC will investigate these issues.

## Competing interests

The authors declare that they have no competing interests.

## Authors' contributions

KCD participated in the design and coordination of the study, developed analysis plans, and drafted the manuscript. JLB participated in the design of the study, assisted with statistical analysis and helped to draft the manuscript. WDE participated in the design and coordination of the study and helped to draft the manuscript. KK preformed the statistical analysis and assisted in drafting the manuscript. All authors read and approved the manuscript.
